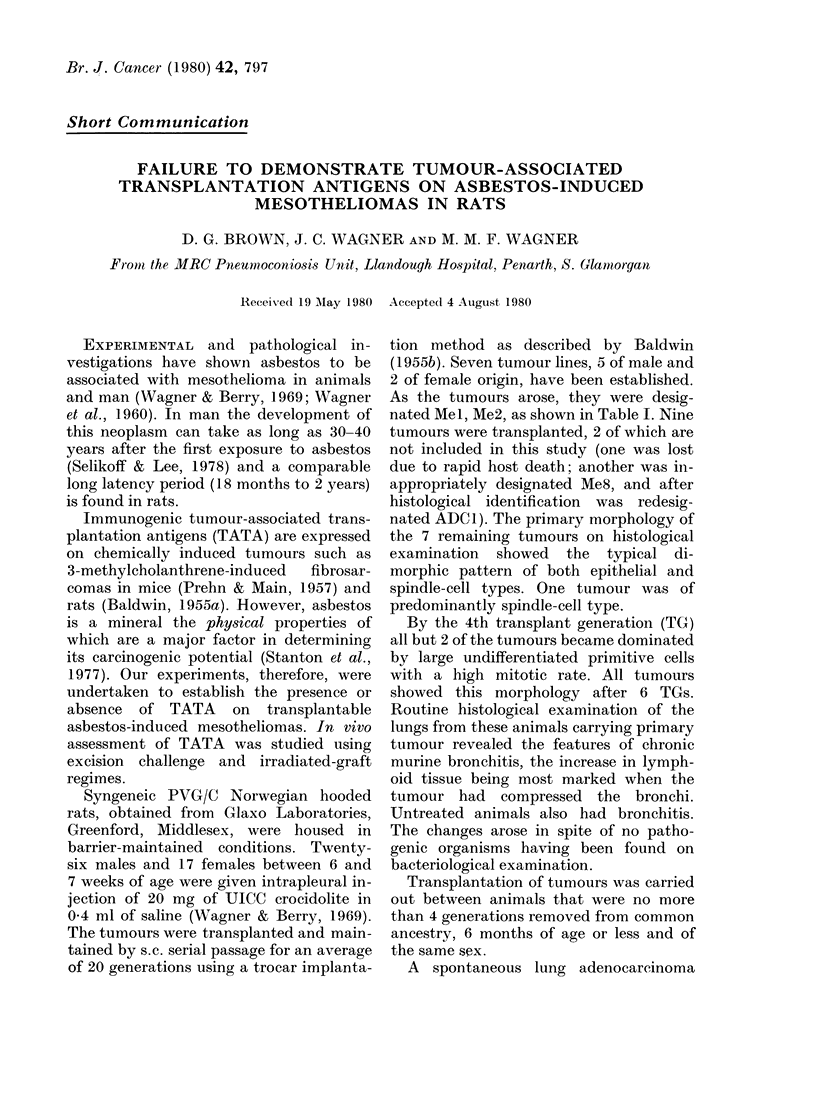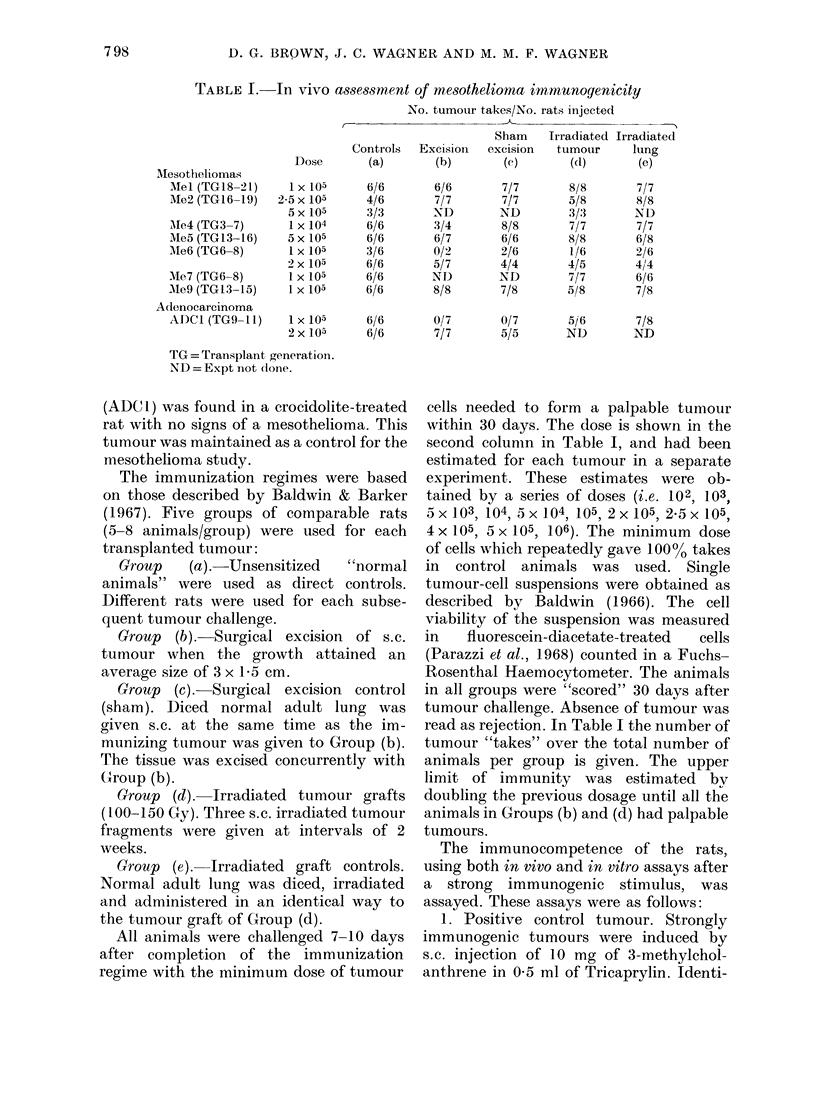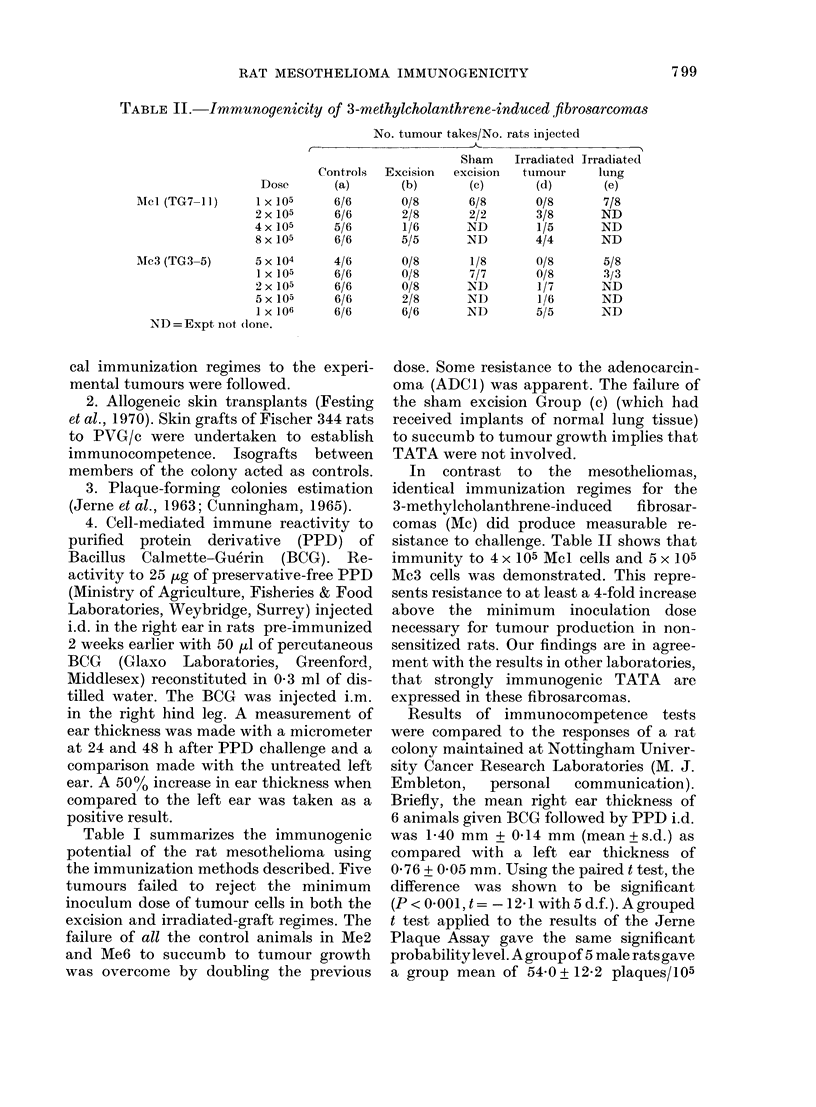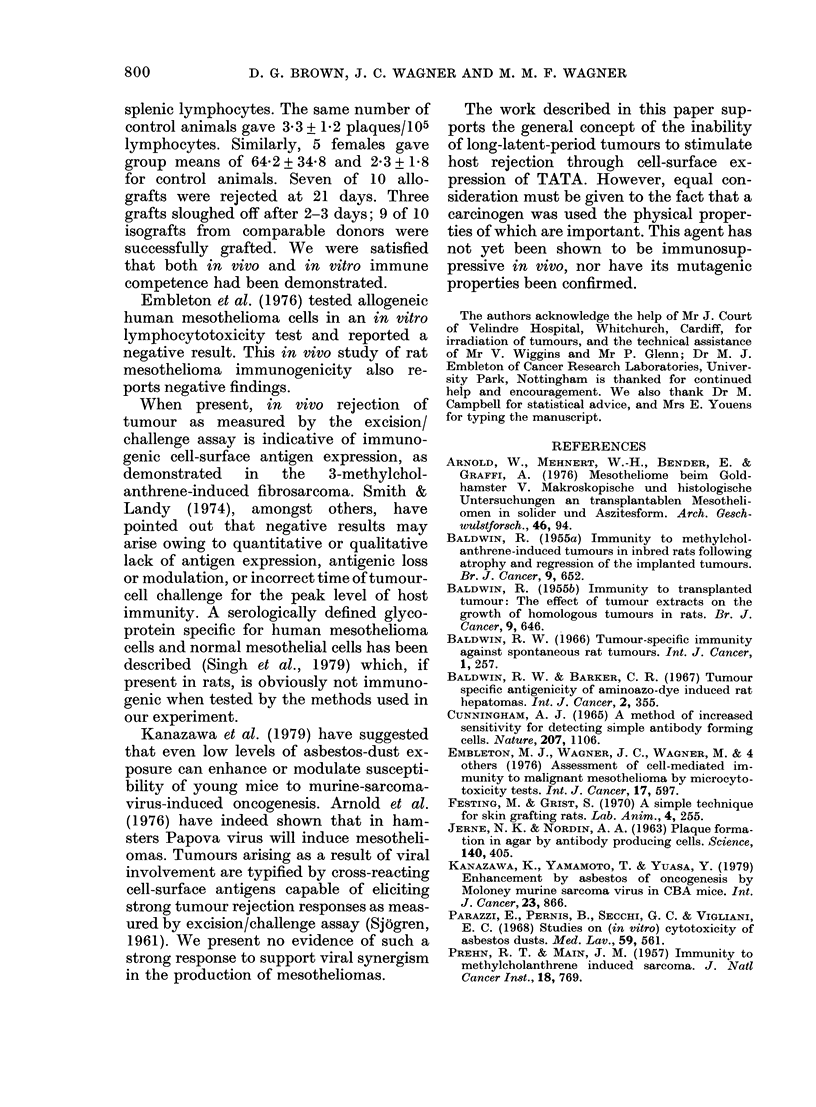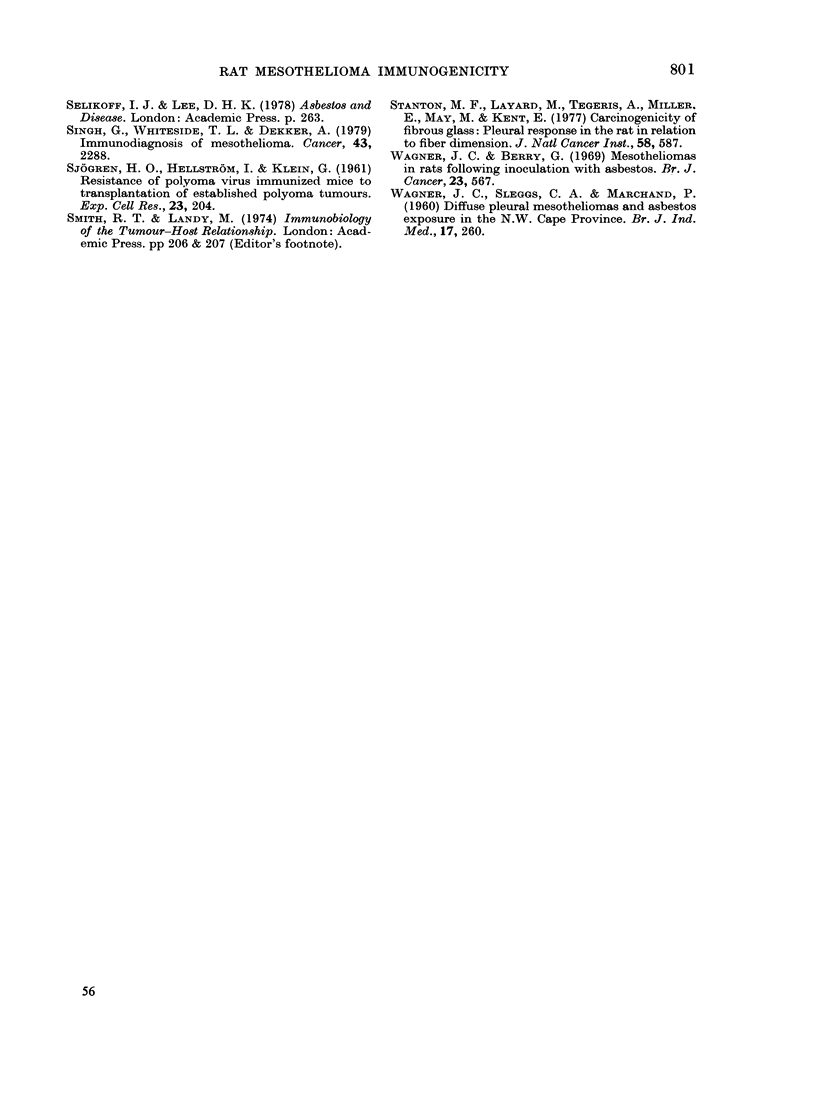# Failure to demonstrate tumour-associated transplantation antigens on asbestos-induced mesotheliomas in rats.

**DOI:** 10.1038/bjc.1980.316

**Published:** 1980-11

**Authors:** D. G. Brown, J. C. Wagner, M. M. Wagner


*Br. J. Cancer* (1980) **42**, 797

***Short Communication***

### FAILURE TO DEMONSTRATE TUMOUR-ASSOCIATED TRANSPLANTATION ANTIGENS ON ASBESTOS-INDUCED MESOTHELIOMAS IN RATS

D. G. BROWN, J. C. WAGNER AND M. M. F. WAGNER

*From the MRC Pneumoconiosis Unit, Llandough Hospital, Penarth, S. Glamorgan*

Received 19 May 1980 Accepted 4 August 1980

EXPERIMENTAL and pathological investigations have shown asbestos to be associated with mesothelioma in animals and man (Wagner & Berry, 1969; Wagner *et al.*, 1960). In man the development of this neoplasm can take as long as 30–40 years after the first exposure to asbestos (Selikoff & Lee, 1978) and a comparable long latency period (18 months to 2 years) is found in rats.

Immunogenic tumour-associated transplantation antigens (TATA) are expressed on chemically induced tumours such as 3-methylcholanthrene-induced fibrosarcomas in mice (Prehn & Main, 1957) and rats (Baldwin, 1955*a*). However, asbestos is a mineral the *physical* properties of which are a major factor in determining its carcinogenic potential (Stanton *et al.*, 1977). Our experiments, therefore, were undertaken to establish the presence or absence of TATA on transplantable asbestos-induced mesotheliomas. *In vivo* assessment of TATA was studied using excision challenge and irradiated-graft regimes.

Syngeneic PVG/C Norwegian hooded rats, obtained from Glaxo Laboratories, Greenford, Middlesex, were housed in barrier-maintained conditions. Twenty-six males and 17 females between 6 and 7 weeks of age were given intrapleural injection of 20 mg of UICC crocidolite in 0·4 ml of saline (Wagner & Berry, 1969). The tumours were transplanted and maintained by s.c. serial passage for an average of 20 generations using a trocar implantation method as described by Baldwin (1955*b*). Seven tumour lines, 5 of male and 2 of female origin, have been established. As the tumours arose, they were designated Me1, Me2, as shown in Table I. Nine tumours were transplanted, 2 of which are not included in this study (one was lost due to rapid host death; another was inappropriately designated Me8, and after histological identification was redesignated ADC1). The primary morphology of the 7 remaining tumours on histological examination showed the typical dimorphic pattern of both epithelial and spindle-cell types. One tumour was of predominantly spindle-cell type.

By the 4th transplant generation (TG) all but 2 of the tumours became dominated by large undifferentiated primitive cells with a high mitotic rate. All tumours showed this morphology after 6 TGs. Routine histological examination of the lungs from these animals carrying primary tumour revealed the features of chronic murine bronchitis, the increase in lymphoid tissue being most marked when the tumour had compressed the bronchi. Untreated animals also had bronchitis. The changes arose in spite of no pathogenic organisms having been found on bacteriological examination.

Transplantation of tumours was carried out between animals that were no more than 4 generations removed from common ancestry, 6 months of age or less and of the same sex.

A spontaneous lung adenocarcinoma

TABLE I.—In vivo *assessment of mesothelioma immunogenicity*

| | Dose | No. tumour takes/No. rats injected | | | | |
|---|---|---|---|---|---|---|
| | | Controls (a) | Excision (b) | Sham excision (c) | Irradiated tumour (d) | Irradiated lung (e) |
| Mesotheliomas | | | | | | |
| Me1 (TG18–21) | $1 \times 10^5$ | 6/6 | 6/6 | 7/7 | 8/8 | 7/7 |
| Me2 (TG16–19) | $2 \cdot 5 \times 10^5$ | 4/6 | 7/7 | 7/7 | 5/8 | 8/8 |
| | $5 \times 10^5$ | 3/3 | ND | ND | 3/3 | ND |
| Me4 (TG3–7) | $1 \times 10^4$ | 6/6 | 3/4 | 8/8 | 7/7 | 7/7 |
| Me5 (TG13–16) | $5 \times 10^5$ | 6/6 | 6/7 | 6/6 | 8/8 | 6/8 |
| Me6 (TG6–8) | $1 \times 10^5$ | 3/6 | 0/2 | 2/6 | 1/6 | 2/6 |
| | $2 \times 10^5$ | 6/6 | 5/7 | 4/4 | 4/5 | 4/4 |
| Me7 (TG6–8) | $1 \times 10^5$ | 6/6 | ND | ND | 7/7 | 6/6 |
| Me9 (TG13–15) | $1 \times 10^5$ | 6/6 | 8/8 | 7/8 | 5/8 | 7/8 |
| Adenocarcinoma | | | | | | |
| ADC1 (TG9–11) | $1 \times 10^5$ | 6/6 | 0/7 | 0/7 | 5/6 | 7/8 |
| | $2 \times 10^5$ | 6/6 | 7/7 | 5/5 | ND | ND |

TG = Transplant generation.
ND = Expt not done.

(ADC1) was found in a crocidolite-treated rat with no signs of a mesothelioma. This tumour was maintained as a control for the mesothelioma study.

The immunization regimes were based on those described by Baldwin & Barker (1967). Five groups of comparable rats (5–8 animals/group) were used for each transplanted tumour:

*Group* (*a*).—Unsensitized "normal animals" were used as direct controls. Different rats were used for each subsequent tumour challenge.

*Group* (*b*).—Surgical excision of s.c. tumour when the growth attained an average size of $3 \times 1 \cdot 5$ cm.

*Group* (*c*).—Surgical excision control (sham). Diced normal adult lung was given s.c. at the same time as the immunizing tumour was given to Group (b). The tissue was excised concurrently with Group (b).

*Group* (*d*).—Irradiated tumour grafts (100–150 Gy). Three s.c. irradiated tumour fragments were given at intervals of 2 weeks.

*Group* (*e*).—Irradiated graft controls. Normal adult lung was diced, irradiated and administered in an identical way to the tumour graft of Group (d).

All animals were challenged 7–10 days after completion of the immunization regime with the minimum dose of tumour cells needed to form a palpable tumour within 30 days. The dose is shown in the second column in Table I, and had been estimated for each tumour in a separate experiment. These estimates were obtained by a series of doses (*i.e.* $10^2$, $10^3$, $5 \times 10^3$, $10^4$, $5 \times 10^4$, $10^5$, $2 \times 10^5$, $2 \cdot 5 \times 10^5$, $4 \times 10^5$, $5 \times 10^5$, $10^6$). The minimum dose of cells which repeatedly gave 100% takes in control animals was used. Single tumour-cell suspensions were obtained as described by Baldwin (1966). The cell viability of the suspension was measured in fluorescein-diacetate-treated cells (Parazzi *et al.*, 1968) counted in a Fuchs–Rosenthal Haemocytometer. The animals in all groups were "scored" 30 days after tumour challenge. Absence of tumour was read as rejection. In Table I the number of tumour "takes" over the total number of animals per group is given. The upper limit of immunity was estimated by doubling the previous dosage until all the animals in Groups (b) and (d) had palpable tumours.

The immunocompetence of the rats, using both *in vivo* and *in vitro* assays after a strong immunogenic stimulus, was assayed. These assays were as follows:

1. Positive control tumour. Strongly immunogenic tumours were induced by s.c. injection of 10 mg of 3-methylcholanthrene in 0·5 ml of Tricaprylin. Identi-

TABLE II.—*Immunogenicity of 3-methylcholanthrene-induced fibrosarcomas*

| | | No. tumour takes/No. rats injected | | | | |
|---|---|---|---|---|---|---|
| | Dose | Controls (a) | Excision (b) | Sham excision (c) | Irradiated tumour (d) | Irradiated lung (e) |
| Mcl (TG7–11) | $1 \times 10^5$ | 6/6 | 0/8 | 6/8 | 0/8 | 7/8 |
| | $2 \times 10^5$ | 6/6 | 2/8 | 2/2 | 3/8 | ND |
| | $4 \times 10^5$ | 5/6 | 1/6 | ND | 1/5 | ND |
| | $8 \times 10^5$ | 6/6 | 5/5 | ND | 4/4 | ND |
| Mc3 (TG3–5) | $5 \times 10^4$ | 4/6 | 0/8 | 1/8 | 0/8 | 5/8 |
| | $1 \times 10^5$ | 6/6 | 0/8 | 7/7 | 0/8 | 3/3 |
| | $2 \times 10^5$ | 6/6 | 0/8 | ND | 1/7 | ND |
| | $5 \times 10^5$ | 6/6 | 2/8 | ND | 1/6 | ND |
| | $1 \times 10^6$ | 6/6 | 6/6 | ND | 5/5 | ND |

ND = Expt not done.

cal immunization regimes to the experimental tumours were followed.

2. Allogeneic skin transplants (Festing *et al.*, 1970). Skin grafts of Fischer 344 rats to PVG/c were undertaken to establish immunocompetence. Isografts between members of the colony acted as controls.

3. Plaque-forming colonies estimation (Jerne *et al.*, 1963; Cunningham, 1965).

4. Cell-mediated immune reactivity to purified protein derivative (PPD) of Bacillus Calmette–Guérin (BCG). Reactivity to 25 $\mu$g of preservative-free PPD (Ministry of Agriculture, Fisheries & Food Laboratories, Weybridge, Surrey) injected i.d. in the right ear in rats pre-immunized 2 weeks earlier with 50 $\mu$l of percutaneous BCG (Glaxo Laboratories, Greenford, Middlesex) reconstituted in 0·3 ml of distilled water. The BCG was injected i.m. in the right hind leg. A measurement of ear thickness was made with a micrometer at 24 and 48 h after PPD challenge and a comparison made with the untreated left ear. A 50% increase in ear thickness when compared to the left ear was taken as a positive result.

Table I summarizes the immunogenic potential of the rat mesothelioma using the immunization methods described. Five tumours failed to reject the minimum inoculum dose of tumour cells in both the excision and irradiated-graft regimes. The failure of *all* the control animals in Me2 and Me6 to succumb to tumour growth was overcome by doubling the previous dose. Some resistance to the adenocarcinoma (ADC1) was apparent. The failure of the sham excision Group (c) (which had received implants of normal lung tissue) to succumb to tumour growth implies that TATA were not involved.

In contrast to the mesotheliomas, identical immunization regimes for the 3-methylcholanthrene-induced fibrosarcomas (Mc) did produce measurable resistance to challenge. Table II shows that immunity to $4 \times 10^5$ Mcl cells and $5 \times 10^5$ Mc3 cells was demonstrated. This represents resistance to at least a 4-fold increase above the minimum inoculation dose necessary for tumour production in non-sensitized rats. Our findings are in agreement with the results in other laboratories, that strongly immunogenic TATA are expressed in these fibrosarcomas.

Results of immunocompetence tests were compared to the responses of a rat colony maintained at Nottingham University Cancer Research Laboratories (M. J. Embleton, personal communication). Briefly, the mean right ear thickness of 6 animals given BCG followed by PPD i.d. was 1·40 mm $\pm$ 0·14 mm (mean $\pm$ s.d.) as compared with a left ear thickness of 0·76 $\pm$ 0·05 mm. Using the paired $t$ test, the difference was shown to be significant ($P < 0{\cdot}001, t = -12{\cdot}1$ with 5 d.f.). A grouped $t$ test applied to the results of the Jerne Plaque Assay gave the same significant probability level. A group of 5 male rats gave a group mean of 54·0 $\pm$ 12·2 plaques/$10^5$

splenic lymphocytes. The same number of control animals gave $3{\cdot}3 \pm 1{\cdot}2$ plaques/$10^5$ lymphocytes. Similarly, 5 females gave group means of $64{\cdot}2 \pm 34{\cdot}8$ and $2{\cdot}3 \pm 1{\cdot}8$ for control animals. Seven of 10 allografts were rejected at 21 days. Three grafts sloughed off after 2–3 days; 9 of 10 isografts from comparable donors were successfully grafted. We were satisfied that both *in vivo* and *in vitro* immune competence had been demonstrated.

Embleton *et al.* (1976) tested allogeneic human mesothelioma cells in an *in vitro* lymphocytotoxicity test and reported a negative result. This *in vivo* study of rat mesothelioma immunogenicity also reports negative findings.

When present, *in vivo* rejection of tumour as measured by the excision/challenge assay is indicative of immunogenic cell-surface antigen expression, as demonstrated in the 3-methylcholanthrene-induced fibrosarcoma. Smith & Landy (1974), amongst others, have pointed out that negative results may arise owing to quantitative or qualitative lack of antigen expression, antigenic loss or modulation, or incorrect time of tumour-cell challenge for the peak level of host immunity. A serologically defined glycoprotein specific for human mesothelioma cells and normal mesothelial cells has been described (Singh *et al.*, 1979) which, if present in rats, is obviously not immunogenic when tested by the methods used in our experiment.

Kanazawa *et al.* (1979) have suggested that even low levels of asbestos-dust exposure can enhance or modulate susceptibility of young mice to murine-sarcoma-virus-induced oncogenesis. Arnold *et al.* (1976) have indeed shown that in hamsters Papova virus will induce mesotheliomas. Tumours arising as a result of viral involvement are typified by cross-reacting cell-surface antigens capable of eliciting strong tumour rejection responses as measured by excision/challenge assay (Sjögren, 1961). We present no evidence of such a strong response to support viral synergism in the production of mesotheliomas.

The work described in this paper supports the general concept of the inability of long-latent-period tumours to stimulate host rejection through cell-surface expression of TATA. However, equal consideration must be given to the fact that a carcinogen was used the physical properties of which are important. This agent has not yet been shown to be immunosuppressive *in vivo*, nor have its mutagenic properties been confirmed.

The authors acknowledge the help of Mr J. Court of Velindre Hospital, Whitchurch, Cardiff, for irradiation of tumours, and the technical assistance of Mr V. Wiggins and Mr P. Glenn; Dr M. J. Embleton of Cancer Research Laboratories, University Park, Nottingham is thanked for continued help and encouragement. We also thank Dr M. Campbell for statistical advice, and Mrs E. Youens for typing the manuscript.

### REFERENCES

Arnold, W., Mehnert, W.-H., Bender, E. & Graffi, A. (1976) Mesotheliome beim Goldhamster V. Makroskopische und histologische Untersuchungen an transplantablen Mesotheliomen in solider und Aszitesform. *Arch. Geschwulstforsch.*, **46**, 94.

Baldwin, R. (1955*a*) Immunity to methylcholanthrene-induced tumours in inbred rats following atrophy and regression of the implanted tumours. *Br. J. Cancer*, **9**, 652.

Baldwin, R. (1955*b*) Immunity to transplanted tumour: The effect of tumour extracts on the growth of homologous tumours in rats. *Br. J. Cancer*, **9**, 646.

Baldwin, R. W. (1966) Tumour-specific immunity against spontaneous rat tumours. *Int. J. Cancer*, **1**, 257.

Baldwin, R. W. & Barker, C. R. (1967) Tumour specific antigenicity of aminoazo-dye induced rat hepatomas. *Int. J. Cancer*, **2**, 355.

Cunningham, A. J. (1965) A method of increased sensitivity for detecting simple antibody forming cells. *Nature*, **207**, 1106.

Embleton, M. J., Wagner, J. C., Wagner, M. & 4 others (1976) Assessment of cell-mediated immunity to malignant mesothelioma by microcytotoxicity tests. *Int. J. Cancer*, **17**, 597.

Festing, M. & Grist, S. (1970) A simple technique for skin grafting rats. *Lab. Anim.*, **4**, 255.

Jerne, N. K. & Nordin, A. A. (1963) Plaque formation in agar by antibody producing cells. *Science*, **140**, 405.

Kanazawa, K., Yamamoto, T. & Yuasa, Y. (1979) Enhancement by asbestos of oncogenesis by Moloney murine sarcoma virus in CBA mice. *Int. J. Cancer*, **23**, 866.

Parazzi, E., Pernis, B., Secchi, G. C. & Vigliani, E. C. (1968) Studies on (*in vitro*) cytotoxicity of asbestos dusts. *Med. Lav.*, **59**, 561.

Prehn, R. T. & Main, J. M. (1957) Immunity to methylcholanthrene induced sarcoma. *J. Natl Cancer Inst.*, **18**, 769.

SELIKOFF, I. J. & LEE, D. H. K. (1978) *Asbestos and Disease*. London: Academic Press. p. 263.

SINGH, G., WHITESIDE, T. L. & DEKKER, A. (1979) Immunodiagnosis of mesothelioma. *Cancer*, **43**, 2288.

SJÖGREN, H. O., HELLSTRÖM, I. & KLEIN, G. (1961) Resistance of polyoma virus immunized mice to transplantation of established polyoma tumours. *Exp. Cell Res.*, **23**, 204.

SMITH, R. T. & LANDY, M. (1974) *Immunobiology of the Tumour–Host Relationship*. London: Academic Press. pp 206 & 207 (Editor's footnote).

STANTON, M. F., LAYARD, M., TEGERIS, A., MILLER, E., MAY, M. & KENT, E. (1977) Carcinogenicity of fibrous glass: Pleural response in the rat in relation to fiber dimension. *J. Natl Cancer Inst.*, **58**, 587.

WAGNER, J. C. & BERRY, G. (1969) Mesotheliomas in rats following inoculation with asbestos. *Br. J. Cancer*, **23**, 567.

WAGNER, J. C., SLEGGS, C. A. & MARCHAND, P. (1960) Diffuse pleural mesotheliomas and asbestos exposure in the N.W. Cape Province. *Br. J. Ind. Med.*, **17**, 260.